# Predictive value of serum miR-21 and miR-122 expression on the efficacy of capecitabine combined with transcatheter hepatic arterial embolization chemotherapy for liver metastasis after colorectal cancer surgery in patients with colorectal cancer and construction and verification of nomograms

**DOI:** 10.3389/fonc.2025.1604994

**Published:** 2025-07-10

**Authors:** Wenfang Ma, Zukuan Chang, Shixing Li, Xiuhua Wang, Guangshao Cao, Youjie Fan

**Affiliations:** ^1^ Department of Interventional Therapy, Xinxiang Central Hospital (the Fourth Clinical College of Xinxiang Medical College), Xinxiang, China; ^2^ Department of Comprehensive Intervention, Henan Provincial People’s Hospital, Zhengzhou, China; ^3^ Department of General Surgery, Xinxiang Central Hospital (The Fourth Clinical College of Xinxiang Medical College), Xinxiang, China

**Keywords:** colorectal cancer, liver metastases, capecitabine, transcatheter hepatic artery embolization chemotherapy, miR-21, miR-122

## Abstract

**Objective:**

To explore the predictive value of serum miR-21 and miR-122 expressions on the efficacy of capecitabine combined with TACE for the treatment of postoperative liver metastasis in colorectal cancer patients, and to construct a nomogram model for verification.

**Methods:**

A total of 252 patients who received this treatment from January 2021 to December 2023 were included in the study. The dataset was randomly split at a 7:3 ratio into a training set (n=181) and a validation set (n=71). Serum levels of miR-21 and miR-122 before treatment were detected and the relationship with clinical pathological characteristics was analyzed. Independent risk factors were screened by multivariate Logistic regression, and a nomogram model was constructed to evaluate efficacy.

**Results:**

In the training set, there were 86 cases with effective treatment and 95 cases with ineffective treatment after operation. Multivariate analysis showed that CEA, high serum miR-21 expression, low miR-122 expression, tumor size, BMI, and age were the independent risk factors for efficacy (*P*<0.05). The nomogram model exhibited C-indexes of 0.809 (training set) and 0.732 (validation set). Additionally, the average absolute errors of the calibration curves were 0.178 and 0.210, respectively. The Hosmer-Lemeshow test result was good. The Receiver operating characteristic (ROC) curve showed that the area under the curve (AUC) of the model in predicting the efficacy was 0.810 (95% *CI*: 0.734-0.885) and 0.731 (95% *CI*: 0.597-0.866) in the training set and the verification set, respectively. The sensitivities and specificities were 0.820, 0.716 and 0.600 and 0.714, respectively.

**Conclusion:**

The expression levels of serum miR-21 and miR-122 have predictive value for the efficacy of liver metastasis after colorectal cancer treatment. The nomogram model has good predictive performance, which can provide a reference for clinical decision-making. Furthermore, the identified predictive value of miR-21 and miR-122 provides a basis for exploring personalized combination therapies with targeted agents in future studies, which may help overcome the limitations of conventional chemotherapy.

## 1 Introduction

Colorectal cancer (CRC) is one of the common malignant tumors worldwide, with high morbidity and mortality (1). Liver metastasis is an important factor for poor prognosis of CRC patients, and about 50% of CRC patients will develop liver metastasis in the disease process (2). Transcatheter arterial chemoembolization (TACE) combined with capecitabine is a commonly used regimen for the treatment of hepatic metastasis after CRC surgery, but the response to this therapy varies among different patients (3). Finding effective biomarkers to predict therapeutic efficacy is crucial for formulating individualized treatment plan and improving patient’s survival rate. MicroRNA (miRNA) is an endogenous non-coding single-stranded RNA, approximately 22 nucleotides in length. It regulates gene expression at the post-transcriptional level by binding to the 3’-untranslated region (3’-UTR) of target mRNA. Studies have shown that miRNA play an important role in the occurrence, development, invasion and metastasis of tumors. Among them, miR-21 and miR-122 are abnormally expressed in a variety of tumors and related to the biological behavior of tumors (4). However, the predictive value of serum miR-21 and miR-122 expressions on the efficacy of capecitabine combined with transcatheter hepatic arterial embolization chemotherapy (TACE) for postoperative liver metastasis in CRC patients has not been clarified (5). A nomogram is a visual prediction model based on multi-factor analysis, which can visually predict the probability of an individual event occurring. It is widely used in the medical field for disease prognosis evaluation and treatment effect prediction. The purpose of this study was to explore the predictive value of serum microRNA-21 (miR-21) and microRNA-122 (miR-122) expressions on the efficacy of capecitabine combined with TACE for the treatment of postoperative liver metastasis in CRC patients, and to construct a nomogram model for verification, so as to provide a reference for clinical decision-making.

## 2 Materials and methods

### 2.1 General information

**Figure 1:**
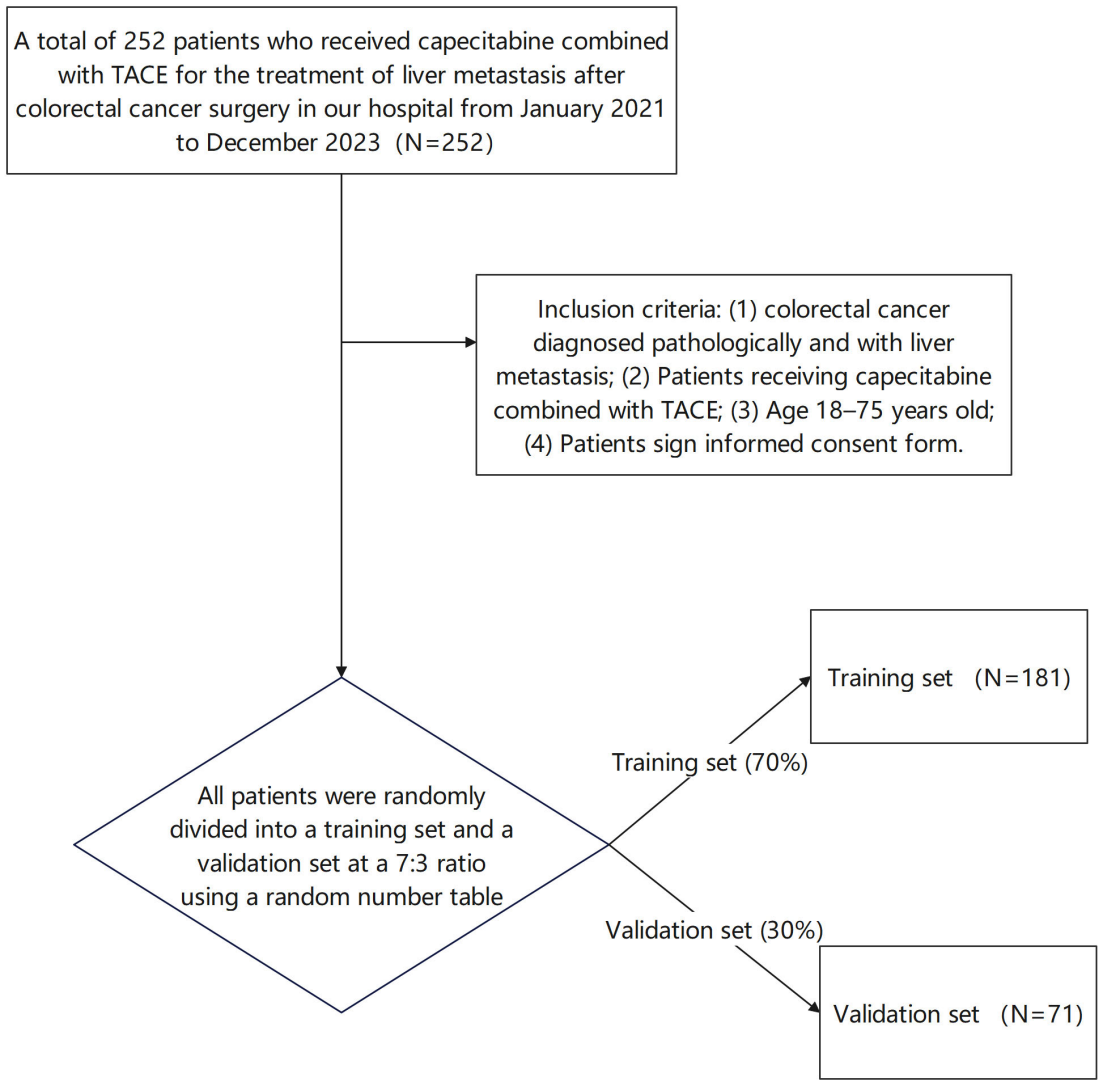
CONSORT flow diagram of patient allocation (Patients were randomly divided into a training set and a validation set at a 7:3 ratio using a random number table).

A total of 252 patients who received capecitabine combined with TACE for the treatment of liver metastasis after colorectal cancer surgery in our hospital from January 2021 to December 2023 were selected. Inclusion criteria (1): colorectal cancer diagnosed pathologically and with liver metastasis (2), Patients receiving capecitabine combined with TACE (3), Age 18–75 years old (4), Patients signed informed consent forms. Exclusion criteria (): Patients with other malignant tumors to avoid confounding effects of concurrent malignancies on treatment response assessment, (2) Severe functional disorders of the heart, liver, kidneys or other vital organs are contraindicated for TACE or systemic chemotherapy. During TACE or systemic chemotherapy, (3) Mental disease precluding informed consent or compliance with the treatment protocol, (4) Other anti-tumor treatments within the past three months, which might influence baseline tumor biology and confound efficacy evaluation of the current regimen. This study was approved by the Hospital Ethics Committee. The Consolidated Standards of Reporting Trials (CONSORT)-style flow diagram of patient allocation is shown in **Figure 1**. Specifically, 181 patients were assigned to the training set, and 71 patients were assigned to the validation set. The randomization process was performed by a statistician who was independent of the study team to ensure objectivity.

### 2.2 Treatment

TACE treatment: Seldinger technique was used for catheterization through femoral artery. The catheter was super-selectively inserted into the inherent hepatic artery or its branches, and hepatic angiography was performed to identify the location, size, number and blood supply of the tumor. Capecitabine (the dose calculated according to the body surface area of the patient) was then mixed with iodinated oil into an emulsion, which was slowly injected into the tumor’s blood supply artery, and the tumor’s blood vessels were embolized with gelatin sponge particles. After the operation, symptomatic support treatments such as liver protection and antiemesis were given. Oral capecitabine treatment: On the first day after TACE treatment, capecitabine at 1000 mg/m was given orally twice a day for 14 consecutive days with a rest of 7 days as one cycle, 4 to 6 cycles in total.

### 2.3 Efficacy evaluation

Treatment efficacy was evaluated according to the Response Evaluation Criteria in Solid Tumors (RECIST) version 1.1. Complete Remission (CR): all target lesions disappeared; Partial Remission (PR): the sum of the longest diameters of the target lesions is reduced by more than or equal to 30% from the baseline; Stable Disease (SD): the sum of the longest diameters of the target lesions is reduced by < 30% or increased by < 20% from baseline; Progressive Disease (PD): The sum of the longest diameters of the target lesions increased by ≥20% from baseline or new lesions appeared. Objective Response Rate (ORR) = (CR+PR)/total cases × 100%; Disease Control Rate (DCR) = (CR+PR+SD)/Total cases ×100%. Patients with CR+PR were included in the effective group and patients with SD+PD were included in the ineffective group. Among the 181 patients included, there were 86 cases in the effective group and 95 cases in the ineffective group.

### 2.4 Detection of serum miR-21 and miR-122

Before the patients received treatment, 5ml fasting venous blood was collected, and serum was centrifuged and stored at -80°C for standby. Total serum RNA was extracted using the Trizol method, and cDNA was synthesized via reverse transcription according to the kit instructions. Real-time quantitative polymerase chain reaction (RT-qPCR) was used to detect the expression levels of miR-21 and miR-122, with U6 as the internal reference gene. Reaction systems and conditions were set according to the kit instructions. The 2-ΔΔ Ct method was used to calculate the relative expression levels of miR-21 and miR-122. The cutoff values for miR-21 (>2.0) and miR-122 (<0.5) were initially derived from prior studies (6). To statistically validate these thresholds in our cohort, we performed receiver operating characteristic (ROC) curve analysis. The Youden index (sensitivity + specificity - 1) was used to determine optimal cutoffs (7). For miR-21, the Youden index-maximizing cutoff was 2.05 (sensitivity 76.5%, specificity 71.8%; AUC=0.78, 95% CI: 0.71–0.85), consistent with the prior threshold of 2.0. For miR-122, the optimal cutoff was 0.48 (sensitivity 73.2%, specificity 68.9%; AUC=0.75, 95% CI: 0.68–0.82), aligning with the established threshold of 0.5. Thus, we retained the published cutoffs (>2.0 for miR-21, <0.5 for miR-122) for clinical translatability.

### 2.5 Collection of clinical pathological data

The clinical and pathological data including age, gender, body mass index(BMI), drinking history, tumor site, tumor size, number of metastases, TNM stage, differentiation degree, serum Carcinoembryonic Antigen (CEA) and carbohydrate antigen 19-9 (CA19-9), serum miR-21 expression level and miR-122 expression level were collected.

### 2.6 Statistical processing

SPSS 25.0 and R 4.0.3 software were used for data analysis. Measurement data were expressed as mean standard deviation (x̄ ± s). Independent sample t test was used for comparison of two groups, and analysis of variance was used for comparison of multiple groups. The count data were expressed as number of cases and percentage (n,%), and the comparison between groups was performed by χ^2^ test. Logistic regression analysis was used to screen the independent risk factors affecting the curative effect of treatment. For continuous variables (CEA, BMI, tumor size, and age), we first assessed their linearity with the outcome using scatter plots and locally weighted scatterplot smoothing (LOESS). No significant nonlinear trends were observed for any of these variables (all *P* for nonlinearity >0.05), so they were treated as linear terms in the logistic regression model. Restricted cubic splines (RCS) were not employed due to the risk of overfitting given the sample size of the training set (n=181), which is below the recommended threshold of ≥200 cases for stable RCS modeling in logistic regression. Application of r software to construct nomograph prediction model based on independent risk factors. The receiver operating characteristic (ROC) curve was used to evaluate the prediction performance of the model, and the Area Under Curve (AUC) and 95% Confidence Interval (CI) were calculated. A calibration curve was used to evaluate the consistency of the model predictions with the actual observations. *P*<0.05 was considered as the difference with statistical significance. For dataset division, patients were first numbered sequentially, and a random number table was used to generate random numbers for each patient. Patients were then sorted by random number values, with the first 70% (n=181) assigned to the training set and the remaining 30% (n=71) to the validation set. The 7:3 training-validation split aligns with established methodologies for predictive model validation, optimizing sample utilization while mitigating overfitting. This study strictly followed the standardized process in the process of data collection to ensure the integrity of clinicopathological data. For a small amount of missing data, complete case analysis method was used, that is, only patients with valid data of all variables were included, and data imputation was not performed. The 252 patients finally included in the analysis had no missing values, ensuring the reliability of the statistical analysis.

## 3 Result

### 3.1 Comparison of clinical pathological characteristics and serum miR-21 and miR-122 expression between the training set and the verification set

After random allocation (7:3 ratio) using a random number table, the training set (n=181) and validation set (n=71) showed no significant differences in baseline characteristics such as age, gender, and most laboratory indicators (*P>*0.05), as shown in **Table 1**.

**Table 1:** Comparison of patient general data characteristics between training set and verification set.

General information		Training set (n = 181)	Validation set (n = 71)	*χ²/t*	*P*
Age (years)		54.33 ± 6.61	55.11 ± 6.21	0.857	0.392
gender	man	101 (55.80)	40 (56.34)	0.006	0.938
	woman	80 (44.20)	31 (43.66)		
BMI (kg/m^2^)		23.42 ± 2.16	23.25 ± 2.31	0.551	0.582
Drinking history	yes	85 (49.96)	30 (42.25)	0.456	0.500
	no	96 (53.04)	41 (57.75)		
Tumor site	colon	88 (48.62)	34 (47.89)	0.011	0.917
	rectum	93 (51.38)	37 (52.11)		
Type of liver metastasis	meanwhile	46 (25.41)	16 (22.54)	0.228	0.633
	Different time	135 (74.59)	55 (77.46)		
Extrahepatic metastasis	yes	35 (19.34)	17 (23.94)	0.661	0.416
	no	146 (80.66)	54 (76.06)		
Tumor size (cm)		4.51 ± 1.36	4.62 ± 1.52	0.558	0.577
Number of metastatic foci (units)		2.98 ± 0.92	3.13 ± 1.02	1.129	0.260
TNM staging	Stage iii	51 (28.18)	18 (25.35)	0.205	0.651
	Stage iv	130 (71.82)	53 (74.65)		
Degree of differentiation	Highly differentiated	48 (26.52)	15 (21.13)	0.791	0.374
	Moderate to low differentiation	133 (73.48)	56 (78.87)		
CEA (ng/ml)		5.83 ± 2.52	6.01 ± 2.41	0.516	0.606
CA19 - 9 (U/ml)		36.32 ± 13.51	37.42 ± 12.26	0.596	0.552
miR-21	High expression	85 (46.96)	40 (56.34)	1.794	0.181
	Low expression	96 (53.04)	31 (43.66)		
miR-122	High expression	98 (54.14)	30 (42.25)	2.885	0.091
	Low expression	83 (45.86)	41 (57.75)		

### 3.2 Univariate analysis of postoperative treatment

There was no significant difference in gender, drinking history, tumor location, type of liver metastasis, and extrahepatic metastasis between the effective group and the ineffective group (*P*>0.05). Significant differences were found in age, BMI, serum miR-21 expression level (relative expression greater than 2.0, considered as a high expression state), miR-122 expression level (relative expression less than 0.5, considered as a low expression state), CA19-9, CEA, differentiation degree, TNM stage, the number of liver metastases, and tumor size (*P*<0.05). Serum miR-21 expression level in the ineffective group was higher than that in the effective group, and miR-122 expression level was lower than that in the effective group. Moreover, the ineffective group had larger tumors, more liver metastases, and later TNM staging (**Table 2**).

**Table 2:** Comparison of general data characteristics between the two groups.

General information		Effective Group (n=86)	Ineffective Group (n=95)	*χ²/t*	*P*
Age (years)		53.33 ± 8.61	57.11 ± 9.21	2.843	0.005
gender	man	49 (56.98)	52 (54.74)	0.092	0.762
	woman	37 (43.02)	43 (45.26)		
BMI (kg/m^2^)		22.12 ± 2.12	23.25 ± 2.31	3.417	0.001
Drinking history	yes	35 (40.70)	50 (52.63)	2.581	0.108
	no	51 (29.30)	45 (47.37)		
Tumor site	colon	42 (48.84)	46 (48.42)	0.003	0.956
	rectum	44 (51.16)	49 (51.58)		
Type of liver metastasis	meanwhile	23 (26.74)	23 (24.21)	0.153	0.696
	Different time	63 (73.26)	72 (75.79)		
Extrahepatic metastasis	yes	12 (13.95)	23 (24.21)	3.045	0.081
	no	74 (86.05)	72 (75.79)		
Tumor size (cm)		4.01 ± 1.21	4.82 ± 1.52	3.939	0.001
Number of metastatic foci (units)		2.81 ± 0.81	3.33 ± 1.12	3.546	0.001
TNM staging	Stage iii	33 (38.37)	18 (18.95)	8.4158	0.004
	Stage iv	53 (61.63)	77 (81.05)		
Degree of differentiation	Highly differentiated	26 (30.23)	22 (23.16)	8.337	0.004
	Moderate to low differentiation	60 (69.77)	73 (76.84)		
CEA (ng/ml)		5.33 ± 2.32	6.11 ± 2.51	2.164	0.032
CA19 - 9 (U/ml)		34.32 ± 12.51	38.62 ± 14.26	2.147	0.033
miR-21	High expression	30 (34.88)	55 (57.89)	9.596	0.002
	Low expression	56 (65.12)	40 (42.11)		
miR-122	High expression	57 (66.28)	41 (43.16)	9.719	0.002
	Low expression	29 (33.72)	54 (56.84)		

### 3.3 Receiver operating characteristic analysis of miRNA cutoffs

ROC curve analysis confirmed the discriminatory power of miR-21 and miR-122 for treatment efficacy prediction. For miR-21, the AUC was 0.78 (95% CI: 0.71–0.85), with a cutoff of >2.0 yielding sensitivity 76.5% and specificity 71.8% (Youden index=0.483). For miR-122, the AUC was 0.75 (95% CI: 0.68–0.82), with a cutoff of <0.5 achieving sensitivity 73.2% and specificity 68.9% (Youden index=0.421).

### 3.4 Logistic regression analysis affecting the curative effect of capecitabine combined with TACE in patients with liver metastasis after colorectal cancer surgery

Factors with statistical differences were included in the Logistic regression model, therapeutic efficacy was taken as the dependent variable (effective group =1, ineffective group =0), and indicators with statistical significance in clinical data (tumor size, number of liver metastases, TNM stage, differentiation degree, CEA, CA19-9, miR-122, miR-21, BMI and age) were taken as the independent variable. The variable assignments are shown in **Table 3**. Logistic regression analysis showed that carcinoembryonic antigen (CEA), high serum miR-21 expression, low miR-122 expression, tumor size, BMI, and age were the independent risk factors for the efficacy of capecitabine combined with TACE in patients with liver metastasis after CRC surgery (*P*<0.05) (**Table 4**).

**Table 3:** Variable assignment method.

Variable	Meaning	Evaluation
X1	Tumor size	continuous variable
X2	Number of metastases	continuous variable
X3	TNM staging	Stage iii =1, stage iv =0
X4	Degree of differentiation	High differentiation =1, medium and low differentiation =0
X5	CEA	continuous variable
X6	CA19 - 9	continuous variable
X7	miR-21	High expression =1, low expression =0
X8	miR-122	High expression =1, low expression =0
X9	BMI	continuous variable
X10	age	continuous variable
Y	Therapeutic efficacy	Valid group =1, Invalid group =0

**Table 4:** Logistic regression analysis affecting the curative effect of capecitabine combined with TACE in patients with liver metastasis after colorectal cancer operation.

Project	B	Standard error	Wald	P	OR	95% Confidence interval
CEA	-0.174	0.076	5.194	0.023	0.84	0.724-0.976
miR-21	0.854	0.429	3.96	0.047	2.35	1.013-5.452
miR-122	1.105	0.432	6.546	0.011	3.02	1.295-7.043
Tumor size	-0.589	0.146	16.265	0.001	0.555	0.417-0.739
BMI	-0.269	0.084	10.293	0.001	0.764	0.648-0.901
age	-0.06	0.021	8.006	0.005	0.942	0.904-0.982

### 3.5 Construction of nomogram prediction model

**Figure 2:**
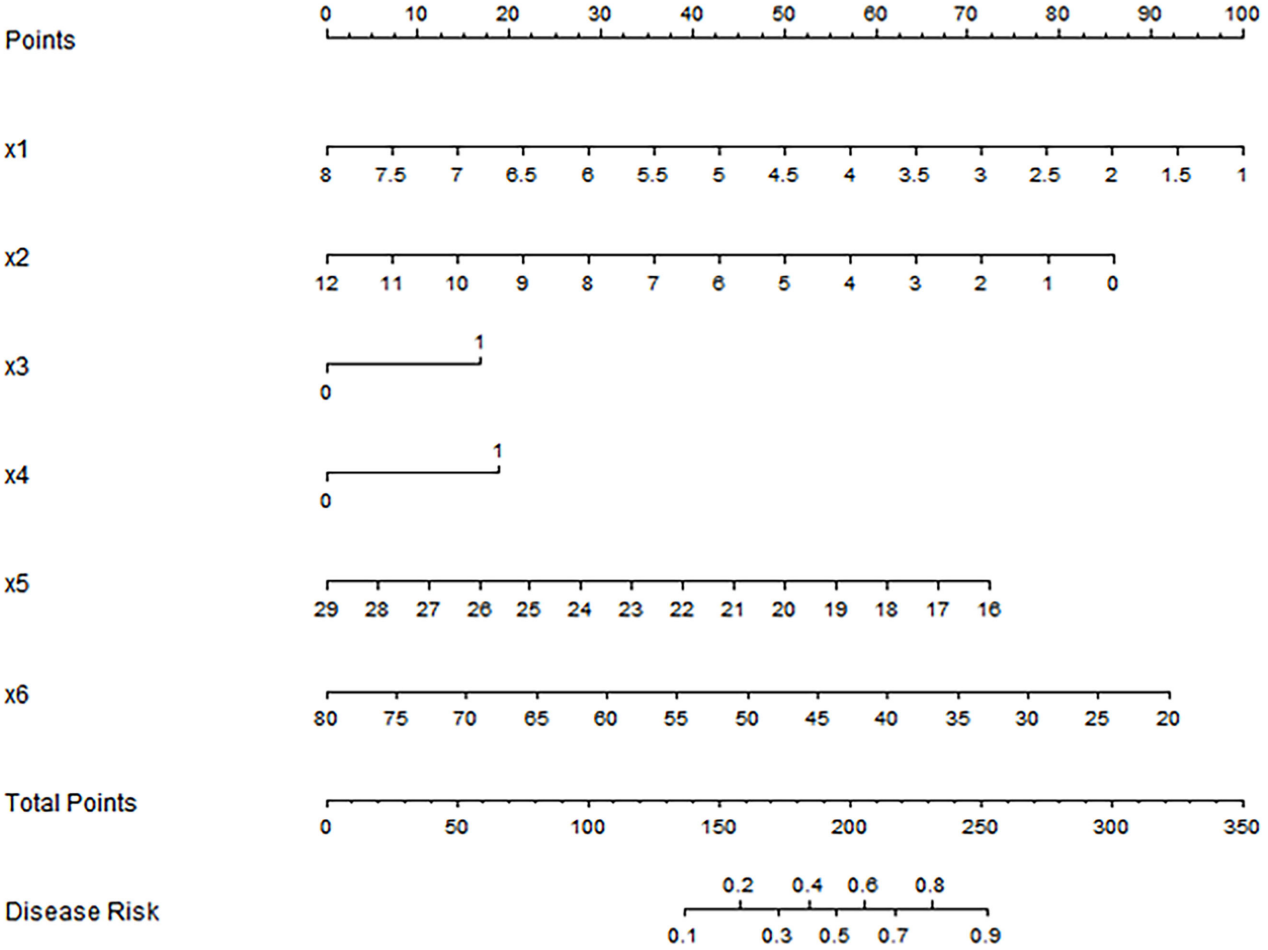
Nomogram prediction model for predicting the efficacy of capecitabine combined with TACE in patients with hepatic metastasis after CRC surgery. X1: tumor size(cm); X2: CEA(ng/ml); X3: high expression of miR-21; X4: low expression of miR-122; x5: BMI(kg/m^2^); X6: Age(years).

Based on the results of Logistic regression analysis, a nomogram prediction model was constructed with R software (**Figure 2**). In the model, factors such as CEA, high serum miR-21 expression, low miR-122 expression, tumor size, BMI, and age are converted into corresponding scores, and the total scores are obtained by accumulating the scores of each factor, so that the probability of ineffective treatment for patients is derived based on the conversion rules of nomogram.

### 3.6 Effect evaluation of nomogram prediction model for predicting the curative effect of capecitabine combined with TACE in patients with liver metastasis after CRC operation

**Figure 3:**
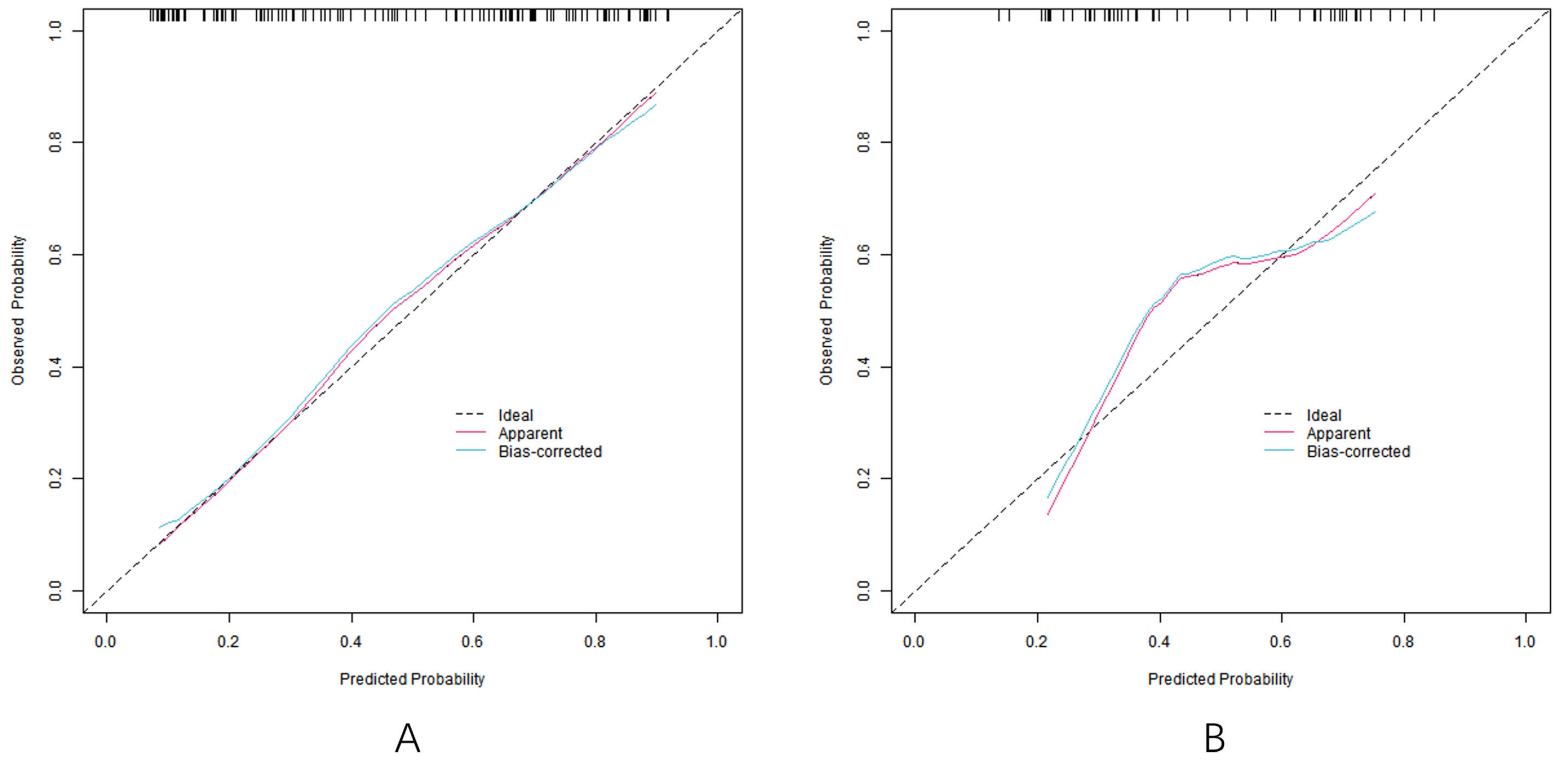
Calibration curve (**A** training set, **B** verification set).

**Figure 4:**
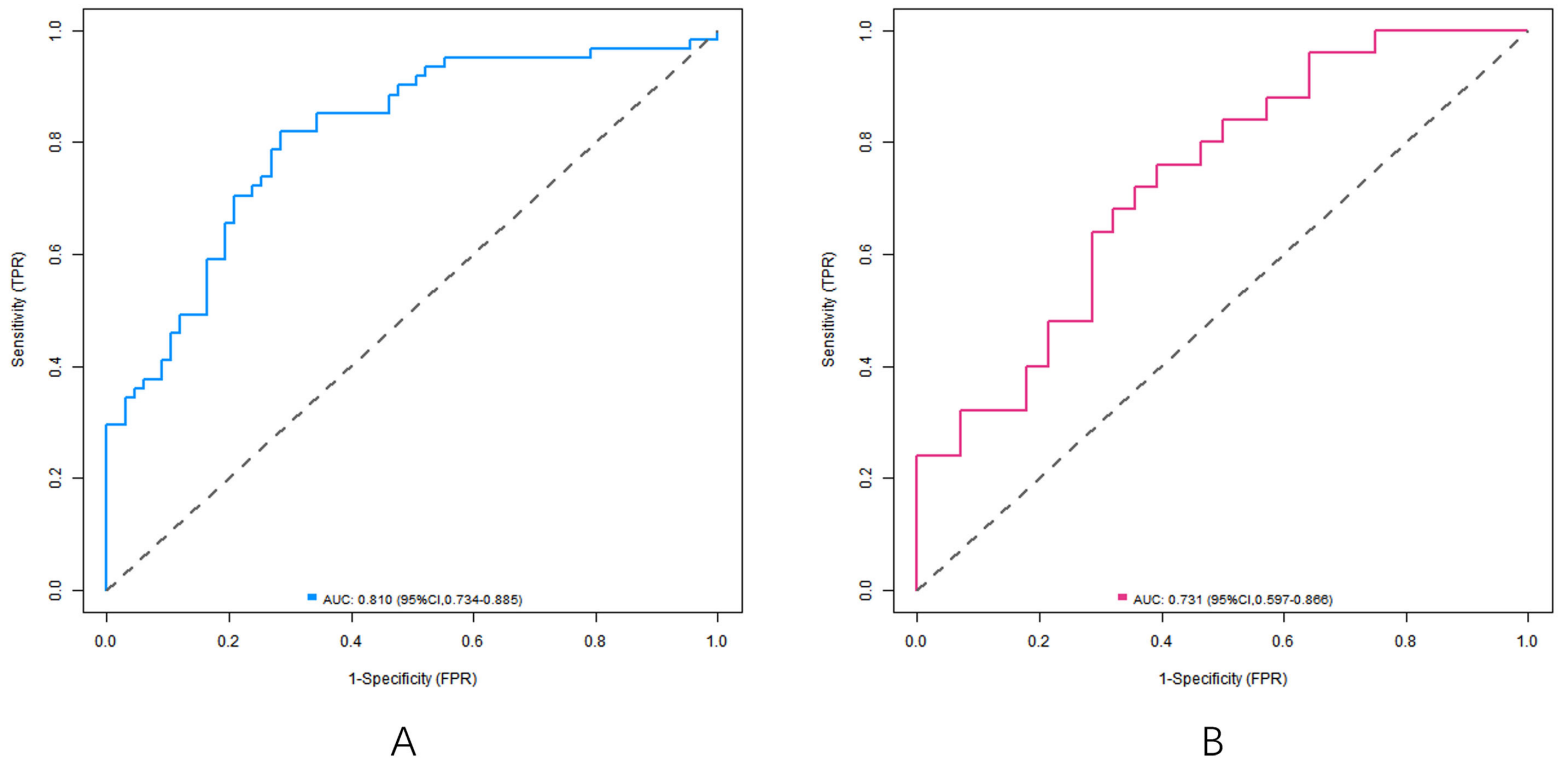
ROC curve (**A** training set, **B** verification set).

In the training set and the verification set, the C-index index of the nomogram model was 0. 809 and 0. 732, respectively, and the average absolute error difference between the predicted value and the actual value shown in the calibration curve was 0.178 and 0.210, respectively. The Hosmer-Lemeshow test results were χ^2^ = 5.108, *P* = 0. 746 and χ^2^ = 11.353, *P* = 0.183, respectively. The ROC curve was shown in the training set and the verification set. The AUC scores of the nomogram model predicting the efficacy of capecitabine combined with TACE in patients with liver metastasis after CRC operation were 0.810 (95% *CI*: 0. 734-0.885) and 0. 731(95% *CI*: 0.597-0.866), and the sensitivity and specificity were 0. 820, 0.716 and 0. 600 and 0.714, respectively. The calibration curve is shown in **Figure 3** and the ROC curve is shown in **Figure 4**.

### 3.7 Decision curve of nomogram prediction model

**Figure 5:**
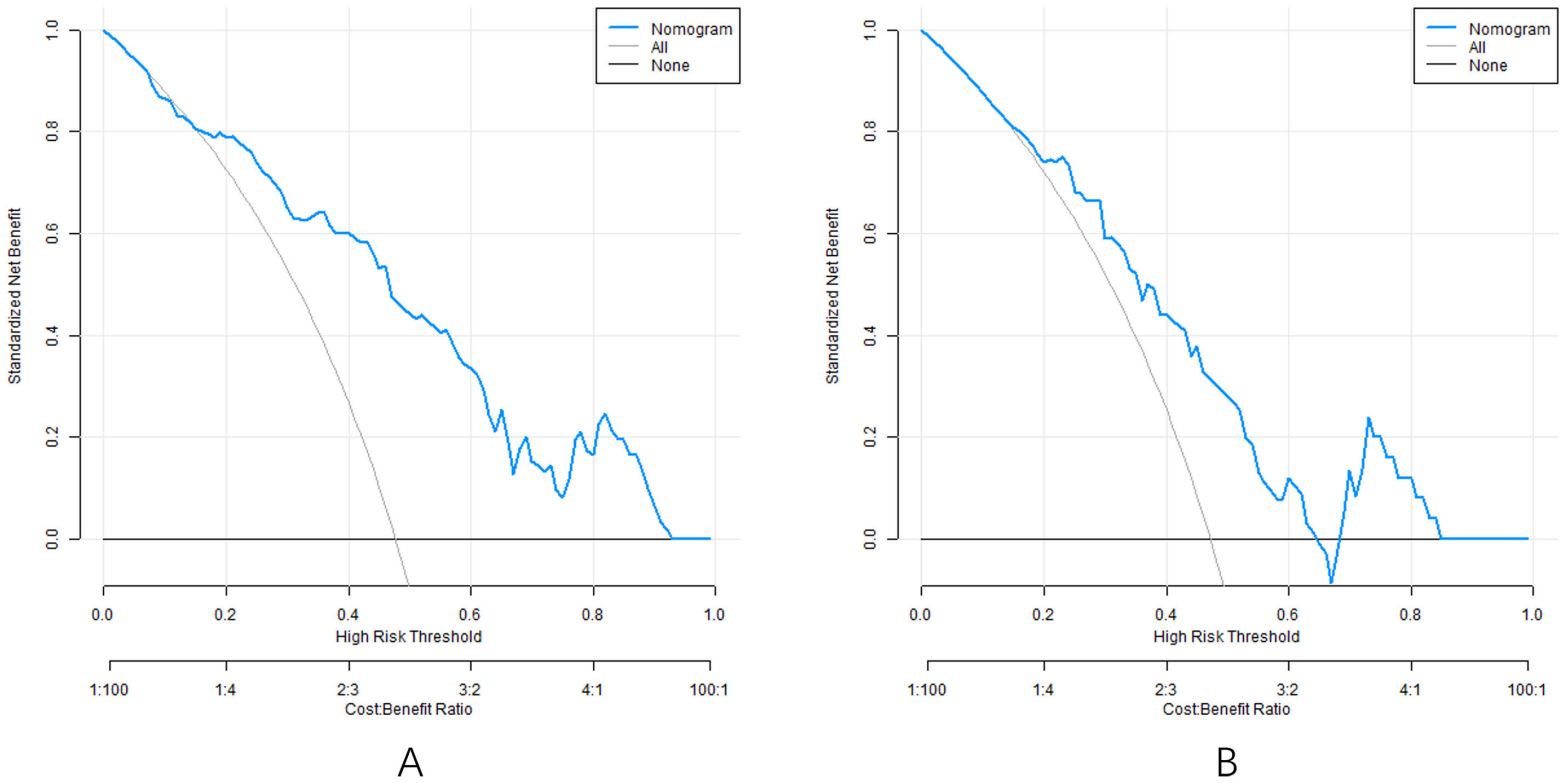
Decision curve (**A** training set, **B** verification set).

Analysis of the decision curve shows that when the threshold probability is about 0.05-0.95, the decision to apply the nomogram model constructed in this study to predict the efficacy of capecitabine combined with TACE in patients with hepatic metastasis after CRC surgery has more clinical benefits than the decision that all are effective or all are ineffective before surgery (**Figure 5**).

## 4 Discussion

This study evaluated the efficacy of capecitabine combined with TACE in patients with post-surgical CRC liver metastasis. We identified that high CEA levels, elevated serum miR-21, low miR-122 expression, tumor size, BMI, and age were independent risk factors for treatment response (*P*<0.05). This achievement has profound significance in clinical practice and tumor research (8). These findings highlight the critical role of pre-treatment monitoring of miR-21 and miR-21 expressions, combined with clinical parameters, in predicting treatment response. As previously reported, such molecular and clinical markers are essential for personalized treatment strategies in oncology. At the time of this study, TACE-based combination chemotherapy remained the mainstream approach for unresectable CRC liver metastases in clinical guidelines, as outlined in the ESMO 2024 Clinical Practice Guidelines (9). Systemic targeted therapies against miR-21/122 (e.g., antisense oligonucleotides or small-molecule inhibitors) were still in preclinical or early-phase clinical trials. Additionally, the translational gap between miRNA biomarker discovery and clinical application of targeted agents is significant, requiring validation of safety, pharmacokinetics, and tumor-specific delivery mechanisms. Thus, the current study aimed to optimize patient selection for standard therapy rather than replace it with unproven targeted approaches.

The cut-off values of miR-21 and miR-122 were initially based on prior literature but further validated in our cohort using ROC analysis. The Youden index-confirmed thresholds (>2.0 for miR-21, <0.5 for miR-122) showed high discriminatory power (AUC>0.75), reinforcing their suitability for clinical application. These thresholds effectively distinguished patients with different treatment responses, as evidenced by the significant correlation with clinical outcomes in univariate analysis. From the perspective of molecular biological mechanism, the high expression of serum miR-21 is closely related to the poor therapeutic effect (10). As a typical pro-cancer miRNA, miR-21 plays a key role in the occurrence and development of a variety of tumors. It inhibits pro-apoptotic gene expression by negatively regulating target genes while activating anti-apoptotic signaling pathways, enabling cancer cells to acquire enhanced proliferative capacity and continuously accumulate, thereby promoting tumor growth (11). Moreover, miR-21 enhances cancer cell invasion and migration by regulating epithelial-mesenchymal transition (EMT)-related factors. This contributes to an increased number of liver metastases. In addition, miR-21 is involved in the regulation of tumor angiogenesis, promoting tumor tissue to form a rich vascular network, providing sufficient nutrients for tumor cells to enable the tumor cells to have more malignant biological behaviors, and greatly reducing the effect of capecitabine combined with TACE treatment (12).

MiR-122 is highly expressed in normal liver tissues and is an important molecule for maintaining normal physiological function of the liver and inhibiting the occurrence and development of tumors. When the serum miR-122 is low in expression, the growth inhibition effect on tumor cells was correlated with attenuated tumor-suppressive effects (13). Studies have shown that miR-122 can target multiple genes related to tumor cell proliferation and invasion, and exert the anti-cancer function by inhibiting the expression of these genes (14). Low-expression miR-122 cannot effectively inhibit the proliferation signaling pathway of tumor cells, leading to accelerated proliferation of cancer cells. At the same time, the invasion and migration ability of cancer cells are enhanced, so that the cancer cells can more easily break through the basement membrane, infiltrate the surrounding tissues and metastasize to a distant place, further aggravating the condition and reducing the effectiveness of treatment (15).

The tumor size, the number of liver metastases, TNM staging, BMI, and age reflect the severity of the disease from the macroscopic characteristics and progression of the tumor. A large tumor volume means a large number of tumor cells. In these cell populations, there may be multiple subpopulations with different biological characteristics, and some of them may have intrinsic resistance to capecitabine and TACE, which is difficult to be completely killed by conventional doses of chemotherapy drugs, thus affecting the therapeutic effect (16). In terms of the number of liver metastases, each metastasis has its own difference in tumor microenvironment and gene expression profile, which increases the complexity and difficulty of treatment and makes it difficult for single capecitabine combined with TACE treatment to produce the ideal therapeutic effect on all metastases (17). In patients with TNM staging, the tumor often has invaded the surrounding tissues and distant organs, and the body’s immune function is severely damaged by the consumption and invasion of the tumor. At this time, the patient’s tolerance to treatment is significantly reduced, and the malignancy of the tumor cells is higher. As a result, the sensitivity to conventional treatment methods is decreased, and the treatment effect is greatly reduced (18).

In clinical practice, this research result provides a key basis for doctors to formulate treatment strategies. For patients with high serum miR-21 expression and low miR-122 expression, high miR-21/low miR-122 expression may identify patients who could benefit from augmented therapies (19).While this study did not incorporate targeted therapies, the identified role of miR-21/122 as predictive biomarkers provides a rationale for future trials exploring biomarker-guided combination strategies. For example, integrating miR-21 inhibitors (e.g., MRG-106 in phase II trials for solid tumors) with TACE and capecitabine could potentially overcome treatment resistance mediated by high miR-21 expression. Additionally, preclinical studies have shown that miR-122 replacement therapy enhances chemosensitivity in liver cancer models, suggesting a synergistic effect when combined with conventional chemotherapy. Such approaches would require multicenter validation and careful evaluation of off-target effects, particularly given the role of miR-122 in normal hepatic function. For patients with tumor size and the number of liver metastases, multidisciplinary consultation (MDT) can be conducted before treatment to comprehensively assess the patient’s body condition and tumor characteristics and formulate personalized comprehensive treatment plan. For example, neoadjuvant chemotherapy is performed before TACE treatment to reduce the tumor volume and the number of metastases, followed by subsequent treatment. Or after TACE treatment, adjuvant radiotherapy is timely given according to the recovery of patients to further control the growth and metastasis of the tumor (20). For patients with TNM staging, in addition to intensive anti-tumor treatment, more attention should be paid to supporting the treatment, improving the nutritional status of patients and enhancing the body immunity to enhance the patient’s tolerance to treatment (21).

Although important results have been achieved in this study, there are still some limitations. The single-center design may introduce selection bias, limiting generalizability. The study lacks external validation due to significant resource constraints, including the complexity of multi-center data coordination, potential variability in miRNA detection protocols across institutions, and the study team’s focus on model optimization during the current phase. These limitations highlight the need for future multi-center studies with diverse populations to validate the nomogram’s generalizability (22). The reason for not performing external validation in this study: 181 patients were selected from January 2021 to December 2023 in this study. A large amount of time and energy were spent on case collection, test indicators, and data analysis during the study cycle. External verification needs to expand the sample source, and contact other medical institutions to obtain data. The coordination process is complex, and it involves issues such as the consistency of test methods, and requires more human, material, and time cost inputs. It may exceed the existing resources and time planning of the research team, resulting in difficulties in conducting external verification. This study focused on exploring the predictive value of serum miR-21 and miR-122 expression on therapeutic efficacy and constructing nomogram model. At the current stage, the team pays more attention to model construction and internal verification. It expects to fully optimize the model and clarify the prediction efficiency in the internal data first, and then consider external verification. The limited research resources will be preferentially focused on the key links, thus laying a foundation for further multi-center research and external verification. Furthermore, integrating deep learning into future external validation efforts could address challenges related to data heterogeneity across multiple centers. For example, transfer learning techniques could adapt the model to different institutional datasets by fine-tuning on target populations while preserving generalizable features. Additionally, deep learning models’ ability to learn robust representations from diverse data sources (e.g., varying miR detection protocols or clinical workflows) may enhance the nomogram’s generalizability. However, such approaches would require standardization of miR quantification methods and clinical data formatting across centers, which remains a critical prerequisite for validating machine learning models in real-world settings. Additionally, resource limitations directly impacted external validation feasibility: Human resources: The research team lacked dedicated personnel for multi-center coordination. Financial constraints: No funding was allocated for inter-institutional collaboration or external data acquisition. Time restrictions: The project timeline could not accommodate prolonged external validation processes. These factors collectively necessitated a focus on internal validation, with external verification deferred to future studies. Additionally, although the relationship between these factors and therapeutic efficacy has been clarified, the upstream and downstream regulatory networks of miR-21 and miR-122 in tumor cells and their intrinsic relationship with tumor size, the number of metastases, and TNM staging need to be further explored, which will help to develop more precise and effective therapeutic targets and therapeutic strategies. Finally, the current study utilized traditional logistic regression for nomogram construction, which may have limitations in capturing complex nonlinear relationships. Although we confirmed linearity for continuous variables using LOESS, the omission of restricted cubic splines (RCS) due to sample size constraints represents a methodological limitation. Future studies with larger cohorts may employ RCS to explore potential nonlinear effects.

In summary, high serum miR-21 expression, low miR-122 expression, tumor size, BMI, and age have significant effects on the efficacy of capecitabine combined with TACE in patients with liver metastasis after CRC surgery. The results of this study offer practical insights for clinicians and highlight potential directions for future tumor research. Future research can rely on multi-center collaborative cooperation and the application of artificial intelligence technology to further deepen the systematic construction and optimization of precision treatment strategies.

## Data Availability

The raw data supporting the conclusions of this article will be made available by the authors, without undue reservation.
